# A case of coinfection with dengue and parainfluenza virus after travel to Indonesia

**DOI:** 10.1093/jtm/taae118

**Published:** 2024-08-31

**Authors:** Fumitaka Momoi, Chie Yamamoto, Ryosuke Hamashima, Keitaro Furukawa, Ryo Araki, Yukiji Yamada, Takaaki Nakaya, Yoko Nukui

**Affiliations:** School of Medicine, Kyoto Prefectural University of Medicine, 602-8566, Kyotoshi, Kamigyo-ku, Kajii-cho, Kawaramachi-Hirokoji, Japan; Department of Infection Control and Laboratory Medicine, Kyoto Prefectural University of Medicine, 602-8566, Kyotoshi, Kamigyo-ku, Kajii-cho, Kawaramachi-Hirokoji, Japan; Department of Infection Control and Laboratory Medicine, Kyoto Prefectural University of Medicine, 602-8566, Kyotoshi, Kamigyo-ku, Kajii-cho, Kawaramachi-Hirokoji, Japan; Department of Infection Control and Laboratory Medicine, Kyoto Prefectural University of Medicine, 602-8566, Kyotoshi, Kamigyo-ku, Kajii-cho, Kawaramachi-Hirokoji, Japan; Kansai Airport Quarantine Station, 549-0001, 1 Senshu Kuko Kita, Izumisano, Osaka, Japan; Department of Clinical Laboratory, Kyoto Prefectural University of Medicine, 602-8566, Kyotoshi, Kamigyo-ku, Kajii-cho, Kawaramachi-Hirokoji, Japan; Department of Infectious Diseases, Graduate School of Medical Science, Kyoto Prefectural University of Medicine, 602-8566, Kyotoshi, Kamigyo-ku, Kajii-cho, Kawaramachi-Hirokoji, Japan; Department of Infection Control and Laboratory Medicine, Kyoto Prefectural University of Medicine, 602-8566, Kyotoshi, Kamigyo-ku, Kajii-cho, Kawaramachi-Hirokoji, Japan

## Abstract

We present a case of coinfection with dengue and parainfluenza viruses, a coinfection that has not been described in the literature to date. This case emphasizes that fever after travel is not always caused by a single disease. Appropriate research on fever sources and infection control measures should be implemented.

An otherwise healthy 20-year-old Japanese man presented to our hospital in Kyoto, Japan, on 21 February 2024, with fever and fatigue. He had travelled to Indonesia 8 days earlier, staying in Yogyakarta and Bali, where he was bitten by mosquitoes several times. He experienced fatigue and back and bilateral joint pain on Day 7 of his stay (Day 0). Upon his return to Japan on Day 1, he developed a fever of 39.7°C and diarrhoea. The rapid diagnostic test (RDT) for dengue fever (SD BIOLINE™ Dengue Duo, Abbott Japan, Tokyo, Japan) administered during airport quarantine was positive for the NS1 antigen. Therefore, he visited our hospital on that day. He had no vital sign abnormalities other than fever and no skin findings other than an acne-like skin rash on his back at the time (Figure 1). Serum real-time reverse transcription-polymerase chain reaction (One Step PrimeScript III RT-qPCR Mix, Takara, Kusatsu, Japan) yielded a Ct value of 26.4 for dengue virus type 2.

**Figure 1:**
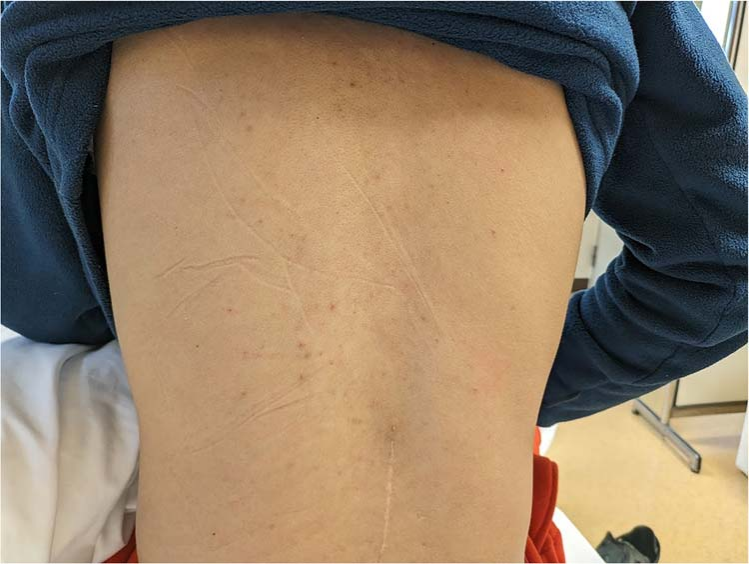
The patient’s skin findings on his first visit (Day 1). During the course, he had no remarkable skin findings other than an acne-like skin rash on his back

Upper respiratory symptoms were unremarkable; however, an additional RDT (FilmArray® Respiratory 2.1 Panel, bioMérieux Japan, Tokyo, Japan) of a nasopharyngeal swab also tested positive for parainfluenza (HPIVs4). He was diagnosed with dengue and parainfluenza virus coinfection.

The patient’s clinical course and platelet counts are shown in Figure 2. He showed rapid thrombocytopenia and was admitted on Day 2; however, no other severe disease indicators, such as shock vital, vomiting, bleeding, fluid retention or hepatomegaly, were observed. With supportive care, including intravenous fluids and antipyretics, his fever resolved on Day 7. His platelet count showed a recovery trend, and he was discharged on Day 8 and recovered steadily thereafter.

**Figure 2:**
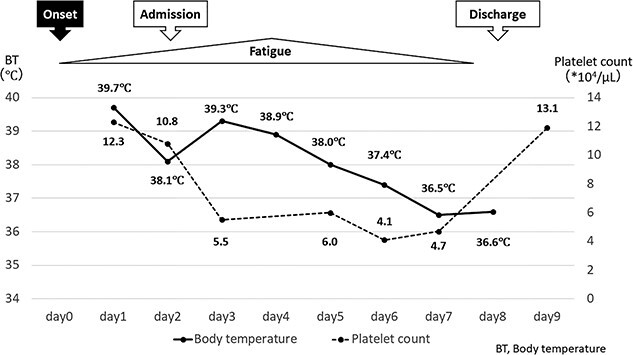
The patient’s clinical course and trend of platelet counts. He showed rapid thrombocytopenia and was admitted on Day 2. He was treated with supportive care because no other severe disease indicators were observed than thrombocytopenia. His fatigue peaked around Day 4–5 and then resolved. His fever resolved on Day 7; however, his platelet count was 41 000/μl on the same day, the lowest during the course. His platelet count showed a recovery trend on Day 8, and he was discharged and recovered steadily thereafter. BT, Body temperature

Dengue fever is one of the most major travel-associated infections; an international multicentre study found that Southeast Asia was the most frequent region of dengue acquisition among travellers from non-endemic countries, accounting for 50.4% of cases.^1^

Coinfections of dengue virus with malaria parasites, chikungunya, Zika, Japanese encephalitis, herpes, measles, influenza virus and SARS-CoV-2 have been reported previously.^2–4^ The impact of these coinfections on the patient’s clinical course is unclear; however, coinfection of dengue virus with SARS-CoV-2 has been suggested to cause severe coronavirus disease 2019 (COVID-19).^3^ Additionally, due to non-specific symptoms, cases of coinfection with SARS-CoV-2 or influenza virus have also been diagnosed after some time of dengue fever diagnosis. Such delays in diagnosis and appropriate infection control measures lead to the spread of nosocomial infections.^4^ Thus, coinfection is an important problem in nosocomial infection control.

Dengue and parainfluenza virus coinfection has not been reported previously. Most parainfluenza virus infections are mild and sporadic; however, they can be severe in infants and immunocompromised adults. Outbreaks can occur in elderly care facilities and wards for patients with severe mental and physical disabilities.

Travel-associated dengue cases dropped dramatically due to reduced global travel during the COVID-19 pandemic.^1^ However, as the COVID-19 pandemic ends, there exists a concern regarding a resurgence in travel-associated dengue cases. Parainfluenza virus infections also decreased due to COVID-19 infection control measures; however, increases have been reported with the easing of these measures.^5^

Fever screening can be conducted in quarantine after overseas travel; however, the fever source is not always a single disease. The symptoms of dengue fever are often nonspecific, and care should be taken to identify coinfections with other diseases. Understanding the prevalence of infectious diseases in a travel area is necessary when obtaining a medical history and conducting a medical examination. If necessary, other infectious diseases should be investigated, and appropriate infection control measures should be taken, even after confirming a diagnosis.

## Data Availability

Raw data were generated by Kyoto Prefectural University of Medicine. The data supporting the findings of this study are available from the corresponding author, Chie Yamamoto, upon request.
